# Staatsfinanzen in der Corona-Krise: Günstige Bedingungen sichern Handlungsfähigkeit

**DOI:** 10.1007/s10273-020-2695-2

**Published:** 2020-07-18

**Authors:** Heinz Gebhardt, Lars-H. Siemers

**Affiliations:** 1 Gneisenaustraße 93, 45472 Mülheim, Deutschland; 2 Fakultät III Wirtschaftswissenschaften, Universität Siegen, Adolf-Reichwein-Straße 2a, 57076 Siegen, Deutschland

**Keywords:** H61, H62, H63

## Abstract

Noch zur Jahreswende 2019/2020 prognostizierten die an der Gemeinschaftsdiagnose beteiligten Wirtschaftsforschungsinstitute für 2020 staatliche Budgetüberschüsse von 21 Mrd. bis 31 Mrd. Euro. Sie gingen von einer Fortsetzung des Aufschwungs aus und erwarteten eine weitere Besserung der staatlichen Finanzlage. Die Staatsfinanzen waren fast ein Jahrzehnt von steigenden Budgetüberschüssen geprägt. Die Corona-Krise verschlechtert die Finanzlage schlagartig; sie wird 2020 zu einem historisch hohen Defizit und einem rasanten Anstieg der Staatsschuldenquote führen. Dank der komfortablen Finanzlage zu Beginn der Corona-Krise und der flexibel ausgestalteten Schuldenbremse verfügt der Staat aber über ausreichende fiskalische Spielräume, um Rettungsschirme und Konjunkturpakete von historischem Ausmaß zu finanzieren.

## Abstract (en)

The general government budgetary position of Germany at the beginning of the coronavirus crisis is advantageous due to extremely favourable conditions of the 2010s. Since 2012, surpluses prevailed, and in 2019 the public debt ratio decreased below 60%. The coronavirus crisis ends this success story dramatically and causes a historically high deficit as well as a rise in the debt ratio to about 77.5% in 2020. This requires consolidating the budget, mainly because age related spending will rise significantly and because an interest rate reversal may involve additional interest payments in the future. To ensure long term debt sustainability, restricting primary expenditures is imperative.

Die fiskalische Handlungsfähigkeit des Staates beruht zum Teil auf glücklichen Umständen; die Konsolidierungsfortschritte vor der Corona-Krise waren weitaus geringer als es auf den ersten Blick scheint. Die imposante Besserung der Staatsfinanzen seit 2010 ist weniger einem strikten Konsolidierungskurs zu verdanken als vielmehr der guten Konjunktur und – in hohem Maße – mehreren Sonderfaktoren, die bereits weggefallen sind, in den kommenden Jahren entfallen oder sich sogar umkehren werden.

Die hohen rezessionsbedingten Defizite infolge der Finanz- und Wirtschaftskrise wurden bis 2012 abgebaut. Seitdem erzielt der Staat Budgetüberschüsse, die bis 2018 stetig stiegen; in den vergangenen vier Jahren beliefen sie sich in Relation zum Bruttoinlandsprodukt (BIP) auf 1,2 % bis 1,9 %. Eine mehrjährige Phase mit Budgetüberschüssen gab es in der Bundesrepublik Deutschland nur einmal, und zwar in den 1950er Jahren zu Beginn der Bonner Republik. Nicht zuletzt aufgrund dieser Überschüsse konnte die im Zuge der Finanz- und Wirtschaftskrise auf 82,4 % emporgeschnellte Staatsschuldenquote deutlich verringert werden. Sie ist 2019 auf 59,8 % gesunken und unterschritt damit erstmals seit 2002 wieder die im Stabilitäts- und Wachstumspakt verankerte Obergrenze. Der zuvor fast kontinuierliche Anstieg der Schuldenquote wurde somit gestoppt und umgekehrt. Die mit der Einführung der Schuldenbremse intendierte Trendwende ist also eingetreten. Die anhaltend hohen Budgetüberschüsse seit 2014 erweckten den Eindruck eines deutlich erweiterten fiskalischen Handlungsspielraums. Aber war die Konsolidierung der Staatsfinanzen vor der Corona-Krise bereits gelungen? Nein! Die Besserung resultiert weitgehend aus der guten Konjunktur, dem historisch niedrigen Zinsniveau, der kalten Progression bei der Einkommensteuer und dem demografischen Zwischenhoch. Die Haushaltsentlastungen in den vergangenen Jahren sind somit zum großen Teil nur temporärer Natur und begründen – anders als es die hohen Überschüsse vor der Corona-Krise suggerieren – keinen nachhaltigen fiskalischen Handlungsspielraum.

## Staatsfinanzen besserten sich konjunkturbedingt

Die deutliche Besserung der staatlichen Finanzlage seit 2010 ist zum Teil dem langen und stabilen Aufschwung zu verdanken. Die Auslastung des gesamtwirtschaftlichen Produktionspotenzials ist seit dem krisenbedingten

**Heinz Gebhardt**, Dipl.-Volkswirt, ist wirtschaftspolitischer Berater und war wissenschaftlicher Mitarbeiter am RWI Essen und Mitglied im Arbeitskreis Steuerschätzungen.

**PD Dr. Lars-H. Siemers** ist wirtschaftspolitischer Berater und Hochschuldozent am Lehrstuhl für Europäische Wirtschaftspolitik an der Universität Siegen.

Tief 2009 kräftig gestiegen; seit 2016 waren die Produktionskapazitäten stärker als normal ausgelastet (BMWi und BMF, 2020). Der mit der konjunkturellen Erholung verbundene Anstieg der Beschäftigung und die deutlich steigenden Löhne und Gewinne ließen das Steuer- und Beitragsaufkommen kräftig sprudeln, und der kontinuierliche Rückgang der Arbeitslosigkeit führte zu sinkenden arbeitsmarktbedingten Ausgaben. Die Konjunkturkomponente, welche die konjunkturellen Einflüsse auf die öffentlichen Haushalte bemisst, ist daher deutlich gestiegen und weist seit 2016 Überschüsse aus (vgl. Abbildung 1). Die öffentlichen Haushalte profitierten von konjunkturellem Rückenwind: die Summe der Konjunkturkomponenten seit 2010 ist positiv. 2020 muss der Staat aber mit erheblichem konjunkturellen Gegenwind zurechtkommen: nach einem lang anhaltenden Aufschwung ist die Konjunktur im Frühjahr 2020 infolge der Corona-Pandemie drastisch eingebrochen, nachdem der Bund und die Länder die sozialen und wirtschaftlichen Aktivitäten massiv einschränkten, um die Ausbreitung der Corona-Pandemie zu verlangsamen und die Kapazitäten des Gesundheitswesens nicht zu überlasten.

Abbildung 1
**Finanzierungssaldo, Konjunkturkomponente und struktureller Finanzierungssaldo des Staates**

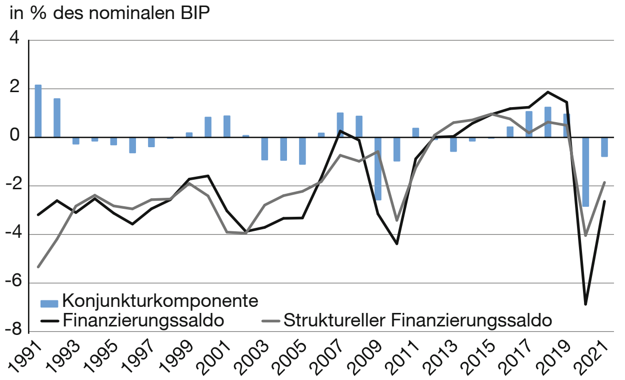

Ist: 1991 bis 2019; Prognose: 2020 bis 2021. Der Finanzierungssaldo 1995 berücksichtigt nicht die Vermögenstransfers im Zusammenhang mit der Übernahme der Schulden der Treuhandanstalt und der Wohnungswirtschaft der ehemaligen DDR (per saldo 119,6 Mrd. Euro).

Quelle: eigene Berechnungen nach Angaben des Statistischen Bundesamtes zur VGR, der Frühjahrsprojektion der Bundesregierung (BMWi und BMF, 2020) und eigener finanzwirtschaftlicher Schätzung.

## Auch strukturell besserte sich die Finanzlage

Auch die um konjunkturelle Einflüsse bereinigte, strukturelle Finanzlage des Staates hat sich in den 2010er Jahren erheblich verbessert. Der Staat hat seit 2012 kontinuierlich strukturelle Überschüsse erzielt (vgl. Abbildung 1). Die strukturelle Besserung ist zum einen dem vom Bund zu Beginn der 2010er Jahre umgesetzten Zukunftspaket zu verdanken, das eine Vielzahl von Einsparmaßnahmen sowie Steuererhöhungen umfasste;[1] zum anderen resultiert sie aus Einsparungen im Haushaltsvollzug von Bund, Ländern und Gemeinden sowie darauf, dass viele Länder die Grunderwerbsteuer erhöhten und die meisten Kommunen ihre Steuern und Abgaben anhoben. Darüber hinaus war das Ausgabenniveau 2010 krisenbedingt überhöht: zum einen durch die Stimulierungsmaßnahmen aus den Konjunkturpaketen, zum anderen durch Vermögenstransfers an die dem staatlichen Sektor zugerechneten Abwicklungsanstalten (Bad Banks) (Deutsche Bundesbank, 2010; Döhrn und Gebhardt, 2013). Für die strukturelle Besserung der Finanzlage waren aber drei weitere Aspekte von erheblicher Bedeutung: die „heimlichen" Steuererhöhungen, die aus der kalten Progression der Einkommensteuer resultieren, das historisch niedrige Zinsniveau und das demografische Zwischenhoch.

## Mehreinnahmen aus der kalten Progression

Das Steueraufkommen ist seit der Finanz- und Wirtschaftskrise stärker gestiegen als das nominale BIP: die Steuerquote nahm von 2010 bis 2019 um 2,2 Prozentpunkte auf 24 % zu und lag damit um 0,5 Prozentpunkte über dem seit der deutschen Wiedervereinigung erreichten Höchstwert aus dem Jahr 2000. Dieser deutliche Anstieg resultiert zum überwiegenden Teil aus den Mehreinnahmen aus der kalten Progression bei der Einkommensteuer. Diese beruhen darauf, dass die Eckwerte des Einkommensteuertarifs seit 2010 nur unzureichend an die Inflation angepasst wurden, sodass auch Einkommenssteigerungen, die lediglich die Inflation ausglichen und somit das Realeinkommen der Steuerpflichtigen nicht steigerten, zu einer höheren Steuerbelastung führten (kalte Progression im engeren Sinne). Auch führten reale Einkommenssteigerungen dazu, dass immer mehr Steuerpflichtige mit höheren Grenzsteuersätzen belastet wurden (kalte Progression im weiteren Sinne). Der Staat absorbierte daher einen zunehmend größeren Teil der privaten Einkommen und profitierte von erheblichen strukturellen Mehreinnahmen.[2]

Da die Mehrbelastungen aus der kalten Progression vielfach durch Anpassungen des Einkommensteuertarifs vermindert

1 Im Rahmen des Zukunftspakets wurden Ausgaben gekürzt, Steuervergünstigungen bei der Energie- und der Stromsteuer eingeschränkt, die Tabaksteuer angehoben sowie die (mittlerweile abgeschaffte) Brennstoffsteuer und die Luftverkehrsteuer eingeführt.

2 Das ifo Institut veranschlagt die durch die kalte Progression von 2011 bis 2016 erzielten Mehreinnahmen auf 70,1 Mrd. Euro; davon 33,5 Mrd. Euro aus der kalten Progression im engeren Sinne und 36,6 Mrd. Euro aus der kalten Progression im weiteren Sinne (Dorn et al., 2017).

werden, können die Mehreinnahmen aus der kalten Progression nicht in vollem Umfang als dauerhaft bzw. strukturell eingestuft werden und überzeichnen die effektiv realisierten Konsolidierungsfortschritte der vergangenen Jahre (Gebhardt und Siemers, 2018). Dies wird in den Regelungen der Schuldenbremse aber nicht berücksichtigt – die Mehreinnahmen aus der kalten Progression werden dort vollständig als strukturelle Besserung eingestuft. Beim Abbau der kalten Progression käme es zu strukturellen Mindereinnahmen, welche die Einhaltung der Schuldenbremse erschweren und gefährden könnten. Seit 2016 wird den Mehreinnahmen aus der kalten Progression indes dadurch entgegengewirkt, dass die Bundesregierung alle zwei Jahre zusammen mit dem Existenzminimumbericht einen Bericht über die Wirkung der kalten Progression im engeren Sinne vorlegt (Steuerprogressionsbericht) und im Zweijahresrhythmus den Einkommensteuertarif an die Inflation anpasst.

## Staat profitierte vom historisch niedrigen Zinsniveau

Der Staat profitierte seit 2010 vor allem vom niedrigen Zinsniveau. Die Zinskonditionen waren für die deutschen Gebietskörperschaften historisch günstig. Das Zinsniveau ist zwar schon seit Ende des vergangenen Jahrhunderts aufgrund struktureller Gründe – wie der Sparschwemme, die zu einem steigenden Kapitalangebot führte – im Trend gefallen; seit der Finanz- und Wirtschaftskrise und der anschließenden Euro-Schuldenkrise ist das Zinsniveau aber primär wegen der außergewöhnlich expansiven Geldpolitik der Europäischen Zentralbank (EZB) sowie der Flucht internationaler Investoren in die als besonders sicher eingeschätzten deutschen Staatsanleihen signifikant gesunken. Während die jahresdurchschnittliche Rendite der zehnjährigen Bundesanleihen vor Beginn der Finanz- und Wirtschaftskrise noch 4,2 % betrug (2007), lag sie 2019 mit -0,3 % auch im Jahresdurchschnitt erstmals im Minus (vgl. Abbildung 2); die reale Rendite ist schon seit 2016 im negativen Bereich. Eine ähnliche Entwicklung ist bei den Länderanleihen zu beobachten (Gebhardt und Siemers, 2020a).

Der implizite Zins der Maastricht-Staatverschuldung (rechnerische Durchschnittsverzinsung) ist von knapp 8 % (1992) auf 4,2 % (2005) gefallen. Danach bewegte er sich bis 2008 auf diesem Niveau seitwärts: die Abwärtstrends der Umlaufrenditen und des impliziten Zinses hatten sich deutlich abgeschwächt (Gebhardt und Siemers, 2020a). Als der implizite Zins im Begriff war, zu steigen, wurde der Konjunkturaufschwung durch die Finanzkrise abrupt beendet. Nicht zuletzt infolge der krisenbedingt sehr expansiven Geldpolitik der EZB ist der implizite Zins dann auf 1,4 % (2019) gefallen (vgl. Abbildung 2); der reale Schuldzins, der 2008 noch 3,8 % betragen hatte, sackte 2017 sogar in den negativen Bereich und verharrt seitdem

Abbildung 2

**Umlaufrendite von Bundesanleihen und Durchschnittszins**

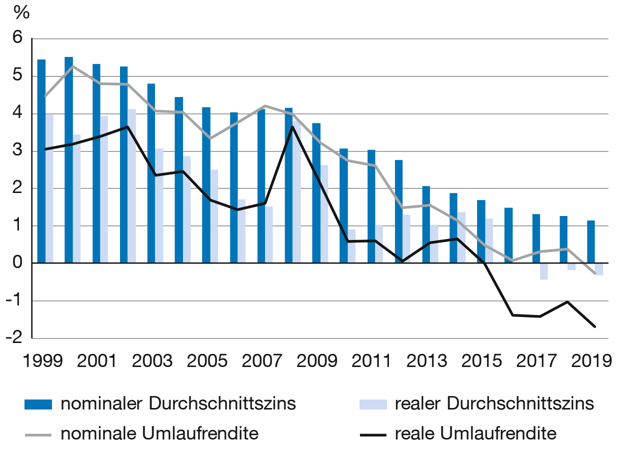

Quelle: Nominale Umlaufrenditen von Bundesanleihen mit einer Restlaufzeit von neun bis zehn Jahren nach Angaben der Deutschen Bundesbank, reale Werte nach eigenen Berechnungen gemäß präziser ex-post Fisher-Gleichung mit Inflation gemäß Verbraucherpreisindex, rechnerischer Durchschnittszins gemäß eigenen Berechnungen nach Angaben der VGR und der Verschuldung des Staates gemäß Maastricht-Abgrenzung nach Angaben der Deutschen Bundesbank.

dort. Seit 2012 kam es so zu erheblichen Einsparungen beim Schuldendienst. Die Zinsausgaben sanken 2019 auf den niedrigsten Stand seit der deutschen Wiedervereinigung. Während der Staat 2007 noch 66,9 Mrd. Euro für den Schuldendienst aufbringen musste, waren es 2019 – trotz der gegenüber 2007 um 453 Mrd. Euro höheren Staatsverschuldung – nur noch 27,5 Mrd. Euro. Nach der Überwindung der Corona-Krise ist indes mit einer Normalisierung der Geldpolitik und somit mit einer sukzessiven Zinswende zu rechnen, die mittelfristig zu einem Anstieg der Zinsausgaben führen wird. Daher überzeichnet die Niedrigzinsphase die Konsolidierungsfortschritte der vergangenen Jahre beträchtlich (Gebhardt und Siemers, 2020a). So sind seit 2007 die Primärausgaben, also die um die Zinsausgaben bereinigten Ausgaben, um 50 % gestiegen – knapp 7 Prozentpunkte stärker als die Ausgaben.

## Demografisches Zwischenhoch entlastete

Die Rentenversicherung profitierte zu Beginn der 2010er Jahre von einem demografischen Zwischenhoch. So stieg der Bevölkerungsanteil der Erwerbsfähigen vorübergehend leicht an, da die geburtenschwachen Nachkriegsjahrgänge das Ruhestandsalter erreichten; hierdurch sind deutlich weniger Rentner hinzugekommen als gestorben, sodass die Rentenversicherung merklich entlastet wurde. Allerdings war diese Entwicklung nur temporärer Natur, denn zum einen sind die Rentenzugänge seit 2014 wieder kräftig gestiegen, wozu die Einführung der abschlagsfreien Rente ab 63 beigetragen hat; zum anderen erreichen ab 2020 die geburtenstarken Babyboomer-Jahrgänge von 1955 bis 1970 das Ruhestandsalter. Der Bevölkerungsanteil der Erwerbsfähigen wird deutlich sinken, während der Anteil der Rentner – trotz des von 2012 bis 2031 sukzessive auf 67 Jahre steigenden Renteneintrittsalters – kräftig zunehmen wird (vgl. Abbildung 3).

Der demografische Wandel, d. h. die aufgrund der niedrigen Geburtenrate sinkende Zahl jüngerer Menschen und die aufgrund der steigenden Lebenserwartung zunehmende Zahl älterer Menschen, stellt das Rentensystem vor eine große Herausforderung. Das sinkende Erwerbspersonenpotenzial wird die Wachstumsperspektiven der deutschen Wirtschaft dämpfen und damit die Bemessungsgrundlage für Steuern und Abgaben schmälern. Gleichzeitig geht die steigende Zahl der Rentner mit einem deutlichen Anstieg der altersabhängigen Ausgaben in den Bereichen Alterssicherung, Gesundheit und Pflege einher[3] und zieht steigende Sozialabgaben nach sich.[4] Mithin muss eine sinkende Zahl von Erwerbstätigen für eine steigende Zahl von Rentnern die soziale Sicherung gewährleisten. Insofern war es im Herbst 2013 kurzsichtig, die Transfers, insbesondere für Erziehungszeiten (Mütterrente) und vorgezogene Renteneintritte (abschlagsfreie Rente mit 63), deutlich auszuweiten; mittel- bis langfristig muss dies über höhere Rentenversicherungsbeiträge finanziert werden (Barabas et al., 2014).

Abbildung 3
**Bevölkerungsanteil der Erwerbsfähigen**

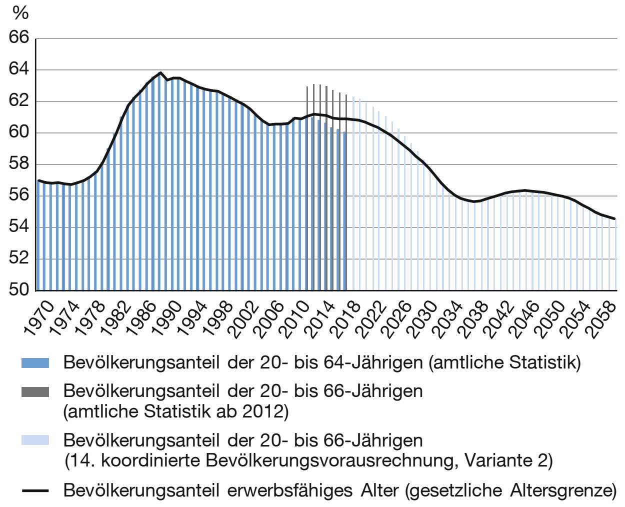

Quelle: eigene Darstellung und Berechnung auf Basis von Destatis (2019), Variante 2.

## Staatsfinanzen im Bann der Corona-Pandemie

Die Besserung der staatlichen Finanzlage in den 2010er Jahren ist somit einer Reihe von Sonderfaktoren zu verdanken. Die dauerhaft verfügbaren fiskalischen Spielräume sind bei weitem nicht so groß, wie es die strukturellen Überschüsse suggerieren. Haushaltsentlastungen aus dem demografischen Zwischenhoch und aus der kalten Progression im engeren Sinne sind bereits weggefallen, und weitere Sonderfaktoren werden in den kommenden Jahren entfallen oder sich sogar umkehren. So führt die Alterung der Bevölkerung schon jetzt zu deutlich steigenden altersabhängigen Ausgaben, und bei einer Normalisierung des Zinsniveaus wäre langfristig mit höheren Zinsausgaben zu rechnen. Zudem profitiert der Staat 2020 nicht mehr von einer guten Konjunktur, sondern muss außerordentlich hohe rezessionsbedingte Haushaltsbelastungen hinnehmen: um die Ausbreitung der Corona-Pandemie zu verlangsamen, wurde in allen großen Volkswirtschaften die Stilllegung von weiten Teilen der Wirtschaft verordnet, was zu einer historischen Rezession führt.

Die Bundesregierung geht in ihrer Frühjahrsprojektion davon aus, dass das BIP nach einem Rückgang um schon 1,9 % im ersten Quartal 2020 im zweiten Vierteljahr um 9 % einbricht, dann aber im dritten und vierten Quartal stark zulegen wird. Sie erwartet somit einen auf wenige Monate begrenzten, aber sehr tiefen Konjunktureinbruch mit einer anschließenden kräftigen Erholung. Sie geht dabei davon aus, dass es nicht zu einem größeren Wiederaufflammen der Pandemie kommt und dass die vom Staat gewährten Liquiditätshilfen und Zuschüsse sowie das erweiterte Kurzarbeitergeld eine Insolvenzwelle verhindern können. Unter diesen Voraussetzungen prognostiziert die Bundesregierung für 2020 einen Rückgang des realen BIP um 6,3 %; dies wäre der stärkste konjunkturelle Einbruch seit dem Zweiten Weltkrieg. 2021 schlägt die erwartete konjunkturelle Erholung dann voll zu Buche: aufgrund von Aufholeffekten und des erwarteten hohen statistischen Überhangs Ende 2020 wird für 2021 mit einem Anstieg des BIP von 5,2 % gerechnet. Da in der Frühjahrsprojektion der Bundesregierung das am 3. Juli 2020 beschlossene Konjunkturpaket noch nicht berücksichtigt werden konnte, dürfte sich die Konjunktur günstiger entwickeln als damals prognostiziert. Die Deutsche Bundesbank (2020b) beziffert in einer ersten groben Abschätzung auf Basis von Standardmodellelastizitäten die BIP-Effekte des Konjunkturpakets auf mehr als 1 % in diesem Jahr und ½ % im kommenden Jahr.

3 Nach Angaben des Tragfähigkeitsberichts 2020 waren 2018 knapp 60 % der Staatsausgaben, also knapp 26% des BIP, demografieabhängig. Je nach Szenario werden sie bis 2060 auf 29 % bis 33 % des BIP steigen (BMF, 2020).

4 Das BMAS (2019) geht im Rentenversicherungsbericht 2019 in der mittleren Variante der Vorausberechnungen davon aus, dass der Beitragssatz in der gesetzlichen Rentenversicherung bis 2024 weiterhin 18,6% beträgt und anschließend steigt (2025: 19,8 %; 2030: 21,2 %; 2033: 22 %).

Diese Prognose ist indes mit Abwärtsrisiken behaftet. Das Wiederhochfahren der wirtschaftlichen Aktivität könnte eine erneute Ansteckungswelle verursachen. Es könnten dann erneute Maßnahmen zur Infektionsbekämpfung in Kraft treten, welche die gesamtwirtschaftliche Nachfrage und Produktion wieder in größerem Umfang beeinträchtigen. Die rezessive Phase würde sich dann nicht auf nur wenige Monate erstrecken und die konjunkturelle Erholung erst später einsetzen. Bei einem U-förmigen Konjunkturverlauf würde der Rückgang des BIP 2020 größer ausfallen als bei dem von der Bundesregierung erwarteten V-förmigen Konjunkturverlauf und wäre mit erheblich höheren volkswirtschaftlichen Kosten verbunden.[5] Der Internationale Währungsfonds (IWF) geht derzeit beim BIP mit -7,8 % von einem stärkeren Einbruch für 2020 aus als die Bundesregierung; für 2021 prognostiziert er indes ebenfalls einen kräftigen Anstieg des BIP in Höhe von 5,4 % (IWF, 2020).

Angesichts des immer noch exponenziellen Wachstums der Pandemie auf globaler Ebene ist davon auszugehen, dass das Herunterfahren und Stilllegen von weiten Teilen der Wirtschaft (weltweit) noch länger anhält und dass es damit weiterhin zu erheblichen Störungen der globalen Liefer- und Wertschöpfungsketten sowie zu reduziertem internationalen Handel kommt.[6] Normalisieren sich die Produktionsprozesse und die gesamtwirtschaftliche Nachfrage nicht, kommt es zu steigenden Einkommens- und Vermögensverlusten sowie Kreditausfällen. Nach dem Konjunktureinbruch käme es dann aufgrund einer Flut von Insolvenzen und einer kräftig steigenden strukturellen Arbeitslosigkeit womöglich noch zu grundlegend ungünstigeren Wachstumsperspektiven (L-förmiger Verlauf des BIP ohne absehbares Ende der Talsohle) und einem noch erheblicheren Anstieg der volkswirtschaftlichen Kosten. Ein solches Worst-Case-Szenario erscheint derzeit indes wenig realistisch.

## Belastung der Staatsfinanzen in historischem Ausmaß

Um die medizinische Versorgung von Corona-Patienten sicherzustellen und die negativen gesamtwirtschaftlichen Auswirkungen der Corona-Pandemie abzufedern, haben der Bund und die Länder Rettungs- und Schutzschirme von historischem Ausmaß aufgespannt. Im März 2020 wurde vom Deutschen Bundestag der größte Nachtragshaushalt in der Geschichte Deutschlands beschlossen, um Mehrausgaben von 122,5 Mrd. Euro und Steuermindereinnahmen von 33,5 Mrd. Euro finanzieren zu können.[7] Zusätzlich wurde wurde im Juni 2020 ein Konjunkturpaket historischen Ausmaßes über 130 Mrd. Euro für 2020 und 2021 geschnürt,[8] was einen zweiten Nachtragshaushalt mit einer Nettokreditaufnahme von 61,8 Mrd. Euro erforderlich machte. Die Nettoneuverschuldung des Bundes beläuft sich mittlerweile auf insgesamt 217,8 Mrd. Euro und umfasst damit über 60 % des ursprünglich für dieses Jahr geplanten Haushalts. Hinzu kommen die Maßnahmen, welche die Länder zur Überwindung der Corona-Krise ergriffen haben, sowie die krisenbedingten Lasten bei den Gemeinden und der Sozialversicherung (Gebhardt und Siemers, 2020b). Ergänzend hat die Bundesregierung den „Wirtschaftsstabilisierungsfonds“ mit Garantieermächtigungen in Höhe von 400 Mrd. Euro und Kreditermächtigungen in Höhe von 200 Mrd. Euro aufgelegt, um Unternehmen, die durch die Corona-Krise in finanzielle Engpässe geraten sind, bei der Sicherung ihrer Liquidität zu unterstützen; diese Maßnahmen haben indes auf den Finanzierungssaldo des Staates in der Abgrenzung des Vertrags von Maastricht, der Änderungen des staatlichen Netto-Finanzvermögens widerspiegelt, nur begrenzt Einfluss.

Die Finanzlage des Staates wird sich durch die Pandemie massiv verschlechtern. Das Budgetdefizit dürfte sich 2020 auf 235 Mrd. Euro bzw. in Relation zum nominalen BIP auf 7,2 % belaufen. 2021 wird es aufgrund der konjunkturellen Erholung, des Auslaufens der Maßnahmen aus den Rettungs- und Schutzschirmen und der geringeren Impulse aus dem Konjunkturpaket deutlich sinken – voraussichtlich auf 89 Mrd. Euro bzw. auf 2,5 % des BIP. Als Folge der Corona-Krise dürfte die Staatsschuldenquote 2020 auf 77½ % hochschnellen (Gebhardt und Siemers 2020b). Deutschland ist zur Bewältigung der hohen pandemiebedingten Haushaltsbelastungen gut gerüstet. Der Bund und die Länder verfügen über eine im internationalen Vergleich sehr gute Bonität am Kapitalmarkt und können sich zu äußerst günstigen Konditionen verschulden. Die Bundesagentur für Arbeit verfügt über Rücklagen

5 Dorn et al. (2020) haben die volkswirtschaftlichen Kosten bei einer Shutdown-Dauer von zwei Monaten auf 255 Mrd. bis 495 Mrd. Euro geschätzt, das BIP fiele um 7,2 bis 11,2 Prozentpunkte; bei drei Monaten steigen die Kosten auf 354 Mrd. bis 729 Mrd. Euro (10,0 bis 20,6 Prozentpunkte Wachstumsverlust).

6 Der IWF (2020) prognostiziert 2020 für viele der wichtigsten Importeure deutscher Waren erhebliche konjunkturelle Einbrüche: -4,9 % (weltweit), -8,0 % (USA), -7,5 % (Euroraum), -12,5 % (Frankreich), -12,8 % (Italien), -12,8 % (Spanien). Dies wird die Exportnachfrage in Deutschland erheblich senken und zu Gewinneinbrüchen bei der Exportindustrie führen.

7 Die Schutz- und Rettungsschirme umfassen eine Vielzahl von Maßnahmen: unter anderem ein Hilfsprogramm zur Sicherung der Liquidität von Unternehmen, Selbstständigen und Freiberuflern; Überbrückungshilfen für Solo-Selbstständige, Kleingewerbetreibende und -unternehmer; die Stundung von Steuerzahlungen und die Anpassung der Vorauszahlungen bei den Ertragsteuern; den erleichterten Zugang zur Grundsicherung, zum Wohngeld und zum Kurzarbeitergeld; Ausgaben zum Ausbau der Intensivkapazitäten in den Krankenhäusern, zur Entwicklung von Präventionsmaßnahmen und zur Erforschung von SARS-CoV-2-Impfstoffen sowie weitere Vorhaben zur unmittelbaren Pandemiebekämpfung. Für einen ausführlichen Überblick vgl. BMWi, 2020.

8 Das Konjunkturpaket enthält eine Vielzahl an Maßnahmen: unter anderem Überbrückungshilfen für kleine und mittelständische Unternehmen (bis zu 25 Mrd. Euro), die temporäre Senkung der Mehrwertsteuersätze (20 Mrd. Euro), die vom Bund finanzierte Senkung der EEG-Umlage beim Strompreis (11 Mrd. Euro) sowie das Vorziehen von geplanten Investitionen (bis zu 10 Mrd. Euro).

von 26 Mrd. Euro und die Krankenkassen (einschließlich Gesundheitsfonds) über Reserven von 30 Mrd. Euro. Zudem ist die Schuldenbremse so flexibel ausgestaltet, dass Bund und Länder in einer außergewöhnlichen Notsituation wie der Corona-Krise sehr umfassende kreditfinanzierte Konjunktur- und Krisenbewältigungspakete auflegen können, wenn diese mit einem Tilgungsplan verknüpft sind.[9] Der Staat verfügt daher 2020 über enorme fiskalische Spielräume, um sich gegen die Corona-Krise zu stemmen.

## Nach der Pandemie warten große Herausforderungen

Die infolge der Corona-Krise emporschnellende Staatsschuldenquote begründet jedoch erheblichen Konsolidierungsbedarf. Dem trägt der Bund unter anderem dadurch Rechnung, dass die krisenbedingte Überschreitung der Regelgrenze für die strukturelle Neuverschuldung in Höhe von 118 Mrd. Euro – wie von der Schuldenbremse vorgegeben – abgebaut werden soll, und zwar von 2023 bis 2042 um jährlich knapp 6 Mrd. Euro. Bei der Einschätzung der finanzwirtschaftlichen Lage muss bedacht werden, dass die großen fiskalischen Handlungsspielräume des Staates in der Corona-Krise günstigen Umständen zu verdanken sind. Von der kalten Progression der Einkommensteuer im engeren Sinne und vom demografischen Zwischenhoch gehen inzwischen aber keine entlastenden Wirkungen mehr aus. Der demografische Wandel führt vielmehr zu stetig steigenden strukturellen Mehrausgaben. Auch ein nennenswerter Rückgang der Zinsausgaben ist nicht mehr zu erwarten; im Gegenteil, langfristig drohen bei einer Zinswende erhebliche Mehrausgaben. Darüber hinaus dürfte die Corona-Krise auch auf EU-Ebene zu höheren Ausgaben führen, um besonders schwer von der Pandemie betroffene Länder, wie Italien oder Spanien, zu unterstützen. All dies begründet weiteren Konsolidierungsbedarf, um die fiskalische Handlungsfähigkeit des Staates zu sichern. Der Konsolidierung der Staatsfinanzen ist daher nach Überwindung der Corona-Krise ein hoher Stellenwert einzuräumen. Um neue, vorrangige Projekte finanzieren zu können, müssen auch Einsparpotenziale bei schon laufenden nachrangigen Projekten zur Gegenfinanzierung ausgeschöpft werden; nur so kann eine nachhaltige Finanzpolitik gelingen und der deutsche Staat seiner wichtigen Rolle als Stabilitätsanker innerhalb der EU gerecht werden.

9 Gleiches gilt bei Deutschlands europäischen Haushaltsverpflichtungen: die allgemeine Ausweichklausel des Stabilitäts- und Wachstumspakts wurde aktiviert, sodass Budgetbeschränkungen temporär keine Anwendung finden und in der Krise höhere Defizite erlaubt sind.

**Title:** ***The German Public Finances in the COVID-19 Pandemic***

**JEL Classification:** *H61, H62, H63*
